# Enhancement of Photothermal Conversion by TiN Nanoparticles-Embedded Black Paint and Applications in Solar Drying of Red Chilli

**DOI:** 10.3390/ma17174393

**Published:** 2024-09-05

**Authors:** Van Thi Thuy Trang, Hoang Thi Hang, Pham Quynh Nhi, Nguyen Thanh Trung, Nhat-Le Bui Dang, Thanh-Lieu Thi Le, Le Thi Cam Nhung, Nguyen Van Nghia, Do Van Can, Hao Van Bui, Loan Le Thi Ngoc

**Affiliations:** 1 Faculty of Natural Sciences, Quy Nhon University, 170 An Duong Vuong, Quy Nhon 55000, Vietnam; trangthuy.251195@gmail.com (V.T.T.T.); hoangthihang@qnu.edu.vn (H.T.H.); quynhnhia4@gmail.com (P.Q.N.); thanhtrung11052005@gmail.com (N.T.T.); lethithanhlieu@qnu.edu.vn (T.-L.T.L.); lethicamnhung@qnu.edu.vn (L.T.C.N.); nguyenvannghia@qnu.edu.vn (N.V.N.); 2 Faculty of Materials Science and Engineering, Phenikaa University, Hanoi 12116, Vietnam; le.dbn19010085@st.phenikaa-uni.edu.vn (N.-L.B.D.); hao.buivan@phenikaa-uni.edu.vn (H.V.B.); 3 Faculty of Engineering and Technology, Quy Nhon University, 170 An Duong Vuong, Quy Nhon 55000, Vietnam; dovancan@qnu.edu.vn

**Keywords:** solar drying, greenhouse, red chilli, black paint, titanium nitride, photothermal effect

## Abstract

This work explores a new application of titanium nitride nanoparticles (TiN NPs) as efficient photothermal materials in enhancing the greenhouse effect. We demonstrate that a simple greenhouse using TiN NPs-embedded black paint boasts several advantages in solar drying technology, which are indicated by the drying of red chilli. In particular, the greenhouse using TiN NPs significantly improves the drying efficiency, which reduces the mass of red chilli by approximately four times and results in dried chilli with a moisture content of 10% within two days. In addition, by conducting long experiments in various environments, we found that the relative humidity can have a predominant role over the temperature in the solar drying of red chilli and observed that the re-adsorption of moisture can take place during the drying process, which prolongs the drying time and reduces the quality of the dried products.

## 1. Introduction

Red chilli is an important spice for daily cuisine worldwide, and serves as a good source of vitamin A, C, potassium, calcium, and folic acid [1]. In recent years, red chilli was recognized as an important agricultural product of Vietnam [2], where it is mostly harvested in the spring time. However, during this season, the market price of red chilli often drops significantly due to its abundant supplies, causing a negative impact on the income of a considerable portion of the population, especially farmers in rural areas. Hence, freshly harvested chilli is often dried and stored until the market price rises in the scarce season. Solar drying is widely adopted for drying red chilli due to its low cost, omni-accessibility, and environmental friendliness [3,4,5,6,7]. In rural areas of Vietnam, open solar drying is a conventional method to dry red chillies, which is accomplished by spreading them on plastic sheets, mats, or cemented floors and exposing them directly to sunlight. Unfortunately, the weather is not favorable for drying red chilli during the harvesting season due to the low temperatures, high relative humidity (RH), and other conditions such as rain and clouds. This is a common situation in many other countries. As a consequence, open solar drying usually takes 7–15 days depending on weather conditions [4]. Moreover, open solar drying often causes contaminations such as dust and microorganisms (e.g., fungi and bacteria), which strongly influence the quality and the lifetime of the dried products [8,9,10]. Hence, many solar drying systems were developed to overcome the drawbacks of the conventional open solar drying [4,5,6,7,8,11,12]. Among various types of solar drying systems, greenhouses (GHs) were demonstrated as the most effective class of solar dryers due to their cost effectiveness and high photothermal conversion efficiency [8,13,14,15,16]. Therefore, GHs are widely adopted for preservation of vegetables and food crops that not only improve the quality of products but also shorten the drying time [7,14,17].

In addition to the development of effective solar drying systems, studying the influence of weather conditions, such as temperature, RH, initial moisture content, solar radiation, and airflow, is essential to achieve insights into the drying process [16,18,19,20,21]. On the one hand, understanding these factors helps in maintaining the quality of dried chilli, preserving essential attributes such as color, flavor, and capsaicin content, which are vital for market acceptance and consumer satisfaction. On the other hand, optimizing these parameters can lead to significant improvements in drying efficiency, reducing energy consumption and drying time, which is crucial for commercial drying operations. Temperature is considered as the most influential factor that determines the drying rate and quality of red chilli [4,21]. Generally, higher temperatures accelerate the drying process by enhancing moisture evaporation. However, excessive temperatures (i.e., up to 65 °C) can degrade the quality of the chilli, particularly its color and capsaicin content. Therefore, a moderate temperature is often recommended to preserve both the drying efficiency and quality. RH is another crucial factor in the drying process. Low relative humidity enhances the drying rate by increasing the vapor pressure deficit, which facilitates moisture removal from the chilli. By conducting experiments using a controlled hot air cabinet dryer which allowed for independent controls of temperature and RH, Getahun and Ebissa revealed that the optimum drying conditions were at a relative humidity of 35% and a temperature of 50 °C for all maturity drying stages [18]. It is commonly believed that temperature is the predominant factor over RH as temperature directly impacts the drying rate by increasing the energy available for moisture evaporation.

Titanium nitride (TiN) was demonstrated as an excellent photothermal material, which is utilized in many applications, such as desalination [22,23], solar heat transducers [24,25,26,27], and solar steam generators [28,29,30,31]. The advantages of TiN rely on its high photothermal conversion, high stability, and strong plasmonic performance that is considered as an alternative candidate for replacing traditional plasmonic materials such as gold and silvers [26,27,32,33]. Importantly, our recent studies demonstrated that TiN can be synthesized from natural ilmenite ore, providing a cost-effective approach for practical applications [34]. Nevertheless, to the best of our knowledge, TiN was not utilized as a photothermal material for solar drying of agricultural products. Hence, in this work, we aim to utilize TiN nanoparticles (NPs) as a photothermal material for solar drying of red chilli. We first design two simple and low-cost GH models for red chilli drying. The first GH model uses commercial black paint as the absorbing material for improving the photothermal conversion efficiency. The second model is developed to improve nighttime drying, which includes a heat box inside. This heat box is made of a metal alloy, whose inner surfaces are coated with TiN NPs-embedded black paint. Our experimental results demonstrate that both models exhibit excellent drying efficiency and significantly reduce the drying time with respect to the conventional open sun drying method. Remarkably, the second model can reduce the mass of red chilli by approximately four times, resulting in dried chilli with a moisture content of 10% within two days. Furthermore, by conducting prolonged drying experiments under various conditions, we observed the re-adsorption of moisture taking place during the drying process, which can prolong the drying time and reduce the quality of the dried product. Furthermore, we found that under certain weather conditions, RH can exhibit a predominant role over the temperature in the solar drying of red chilli. The results obtained from our work bring not only new greenhouse designs for practical applications, especially for farmers in rural areas, but also new insights into the drying behavior of red chilli.

## 2. Materials and Methods

### 2.1. Synthesis of TiN NPs

TiN NPs were synthesized by NH_3_ treatment of commercial P25 TiO_2_ nanoparticles (Evonik, Darmstadt, Germany) at a temperature of 800 °C in a home-built reactor as described elsewhere [34,35]. For each experiment, 500 mg of the powder were loaded into a ceramic boat and placed horizontally in the reactor. Before heating, the reactor was evacuated to a base pressure of 2 × 10^−2^ mbar using a mechanical pump. Then, a constant N_2_ flow of 500 sccm was introduced into the reactor, while keeping a constant pump speed. In the next step, the reactor was heated with a heating rate of 20 °C·min^−1^. When the treatment temperature was reached, the reactor was evacuated again to the pressure of 2 × 10^−2^ mbar. Thereafter, a flow of 200 sccm of NH_3_ (99.9 vol.%) was introduced to the reactor. The treatment time was fixed at 2 h. After this treatment step, the reactor was cooled down to room temperature under a constant N_2_ flow of 500 sccm.

### 2.2. Materials Characterization

The surface morphology of the synthesized materials was characterized by using a HITACHI S-4800 field emission scanning electron microscope (FE-SEM, HITACHI, Tokyo, Japan). SEM images were taken at an operating voltage of 5.0 kV and with a magnification of 50,000 times. The XRD diffractograms of the samples were investigated by a D2 phaser diffractometer (Brucker, Rheinstetten, Germany), equipped with an X-ray source generating X-rays with a wavelength of 1.54 Å (Cu Kα). The data were collected in the 2θ range of 20–80°. The optical reflectance spectra and UV-Vis absorption spectra were measured using a JASCO-V-770 spectrophotometer (JASCO, Hachioji, Japan). The data were recorded in the wavelength range of 350–800 nm.

### 2.3. Preparation of Black-Painted Plates

Flat Al-Zn alloy plates with a thickness of 0.5 mm were used as substrates. The upper side of the plates was painted with commercial black paint (Expo Alkyd Oil Paint, Vung Tau, Vietnam). The thickness of the painting layer is approximately 0.1 mm.

### 2.4. Preparation of the Heat Box

The heat box was made of Al-Zn alloy with dimensions of 20 cm × 15 cm × 20 cm. This box was put inside the first GH when used. The inner surfaces of the box were painted with two layers of absorbing materials. The inner layer, i.e., the buffered layer, was the commercial black paint (Expo Alkyd Oil Paint, Vietnam). The outer layer was a mixture of the commercial black paint and TiN NPs. Each unit of the mixture was prepared by mixing 10 mL of the black paint with 50 mL of acetone (99.5%, Fisher, Schwerte, Germany, CAS number 67-64-1) and 2 g of the synthesized TiN NPs. Both layers were coated by roll-coating and dried naturally at room temperature. A commercial W-filament light bulb (50 W, Dien Quang JSC., Thu Duc, Vietnam) was used as the light source, which was installed inside the box. When the lamp is turned on, the box is heated, which functions as a heat source inside GH.

### 2.5. Construction of the Greenhouse

**Figure 1:**
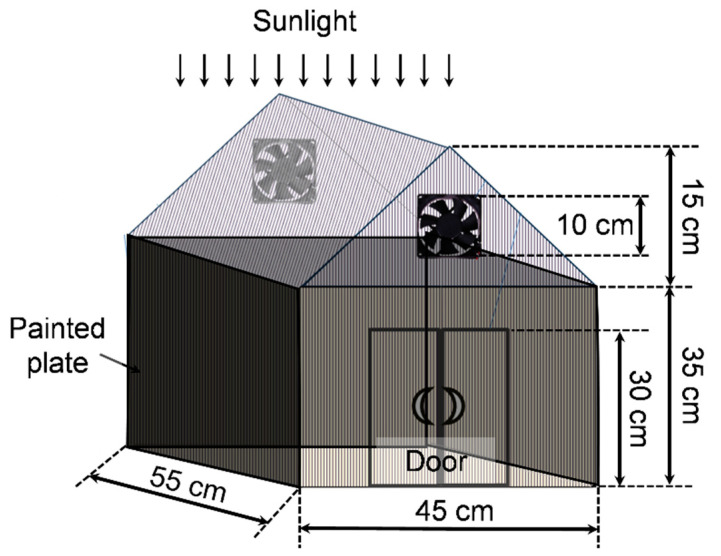
A schematic drawing of the greenhouse containing three black-painted metal plates on three walls.

The greenhouse (GH) was constructed using an aluminum frame with dimensions illustrated in Figure 1. The walls and roofs of GH were made of hollow polycarbonate sheets (specific weight of 1.2 kg·m^−2^ with a thickness of 5.5 mm). This structure provided several benefits, including excellent thermal isolation, light weight, superior rigidity and impact resistance, as well as weather and UV resistance. Two fans were installed on two opposite sides of the GH for ventilation. A door was made on a wall of GH for loading and unloading samples.

### 2.6. Solar Drying of Red Chilli

For each experiment, 500 g of fresh red chilli collected from a local farm (Binh Dinh Province, Vietnam) was uniformly distributed in a stainless-steel basket. The weight of the basket and red chilli was measured by an electronic balance with an accuracy of 0.01 g. The weight was monitored and read remotely by using a digital camera installed inside GH. The temperature and RH inside GH were monitored using a wireless hygrometer-thermometer (Chuangjia, 5558RFTH, Changzhou, China). The temperature inside was additionally measured by CENTER-300 digital thermometers equipped with K-type thermocouples (Center Technology Corp., New Taipei City, Taiwan), which showed a good agreement with the temperature indicated by the hygrometer–thermometer. Another hygrometer–thermometer was used to monitor the ambient temperature and RH. The experiments were conducted in three different time periods, i.e., December–March, April–July, and August–November, in 2023 and 2024, and at the location with the latitude and longitude of 13.7820° N and 109.2193° E, respectively.

### 2.7. Calculations of Moisture Content and Drying Rate

The moisture content of chilli at any drying time was calculated following the formula [18,36,37]:

(1) $$M_{d}=\frac{w\left(t\right)-w_{d}}{w_{d}},\;\mathrm{and}\;M_{d}=\frac{M_{w}}{1-M_{w}},$$

where $M_{d}$ and $M_{w}$ are dry basis and wet basis moisture content (%), respectively, $w\left(t\right)$ is the mass of wet material at time *t* (in g), and $w_{d}$ is the mass of dry material (in g). The moisture ratio *MR* (dimensionless) is calculated as:

(2) $$MR=\frac{M_{0}-M_{e}}{M_{w}-M_{e}},$$

where $M_{0}$ is the initial moisture content (%, wet basis) and $M_{e}$ is the equilibrium moisture content (%, wet basis). The drying rate (*DR*) is calculated following the formula:

(3) $$DR=\frac{M_{t}-M_{t+∆t}}{∆t},$$

where $M_{t}$ is the moisture content at the time *t* (%, wet basis), $M_{t+∆t}$ is the moisture content at the time t+∆t(%, wet basis), and ∆t is the time interval.

For red chilli, it was reported that the initial wet basis moisture content is approximately 80% and the equilibrium moisture content is 10% [19]. Therefore, in our calculations, these values were used to determine the wet basis moisture content and the drying rate.

## 3. Results and Discussion

### 3.1. Morphology, Crystalline Structure and Light Absorption of TiN NPs

**Figure 2:**
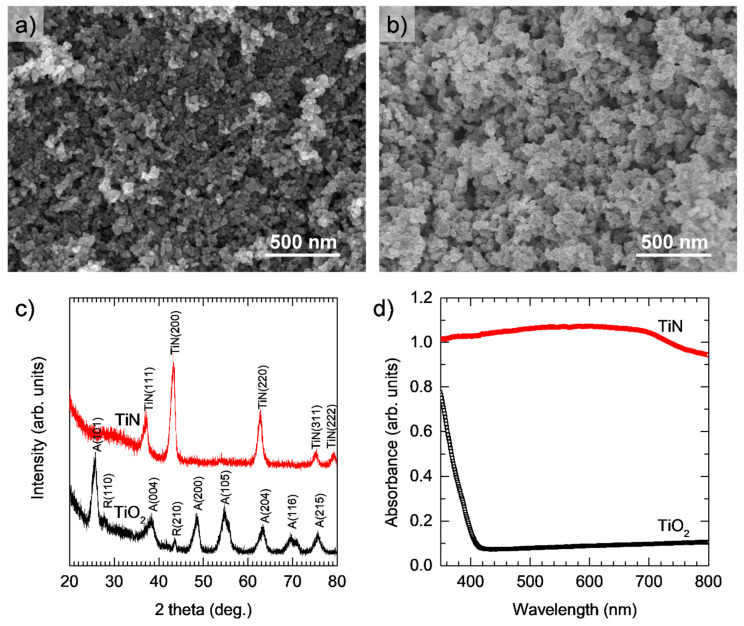
SEM images of (**a**) P25 TiO_2_ NPs and (**b**) TiN NPs obtained by NH_3_ treatment of P25 TiO_2_ at 800 °C; (**c**) XRD diffractograms and (**d**) UV-Vis absorption spectra of P25 TiO_2_ and TiN.

Figure 2a is a SEM image of the initial P25 TiO_2_. According to the supplier, the average particle size is in the range of 20–25 nm. The SEM image of the material obtained after the NH_3_ treatment at 800 °C for 2 h is shown in Figure 2b. By comparing the two images, we can see that the NH_3_ treatment at 800 °C did not cause any significant change in particle size. However, the treatment alters entirely the crystalline structure as well as the light absorption properties of the material. In particular, Figure 2c demonstrates that the crystalline structure of P25 TiO_2_, which consists of both anatase and rutile phases, is completely converted into the cubic crystalline structure of TiN [34]. At the same time, the UV-Vis spectra in Figure 2d show that P25 TiO_2_ only absorbs light in the UV region. This is common for P25 TiO_2_, which arises from its large bandgap (i.e., ~3.2 eV) [38]. In contrast, TiN strongly absorbs light in the entire visible region, which is similar to the effect observed for other nanostructured TiN materials [34,35]. The results suggest that TiN is highly suitable for solar energy harvesting.

### 3.2. Light Absorption and Photothermal Conversion of TiN-NPs-Embedded Black Paint

**Figure 3:**
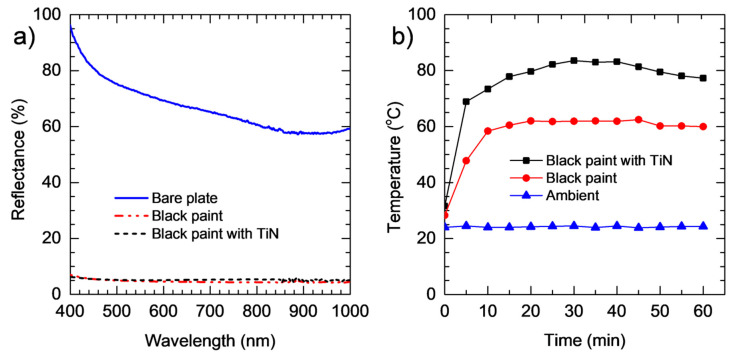
(**a**) Reflectance spectra of the bare Al-Zn alloy plate and the plates painted with a thin layer of black paint without and with TiN NPs. (**b**) Surface temperatures of the plate painted with a layer of black paint (●) and TiN-NPs-embedded black paint (◼) under the illumination of the light generated by a W-filament bulb (50 W). The ambient temperature (▲) is also plotted for comparison.

Figure 3a presents the reflectance spectra of the bare metal plate and the plates painted with a thin layer of black paint without and with TiN NPs. The spectrum of the bare metal plate shows a reflectance of approximately 60% or higher in the entire measured wavelength range. Both the painted plates, however, show a reflectance of only ~5% in the entire spectral region. This indicates an outstanding light absorption performance and implies a high photothermal conversion efficiency of the painted layer.

Although the reflectance spectra in Figure 3a show identical behavior, the photothermal conversion of the black paint embedded with TiN NPs is remarkably higher. This is indicated in Figure 3b, in which the surface temperatures of the two painted plates under the illumination of the light generated by a W-filament bulb are shown. We note that the measurements were conducted indoors with air conditioning; hence, the ambient temperature was kept at ~25 °C. The results show that in both cases, the temperature increases rapidly in the first 15 min, followed by saturation with increasing the illumination time. In the saturating regime, the surface temperature of the black-painted plate without TiN NPs varies between 60 and 62.5 °C, whereas the temperature of the plate with TiN NPs is in between 77.3 and 83.6 °C. Therefore, we can conclude that the inclusion of TiN NPs can improve the photothermal conversion of the black paint.

### 3.3. Drying of Red Chilli by Sunlight

#### 3.3.1. Open Sun Drying of Red Chilli

**Figure 4:**
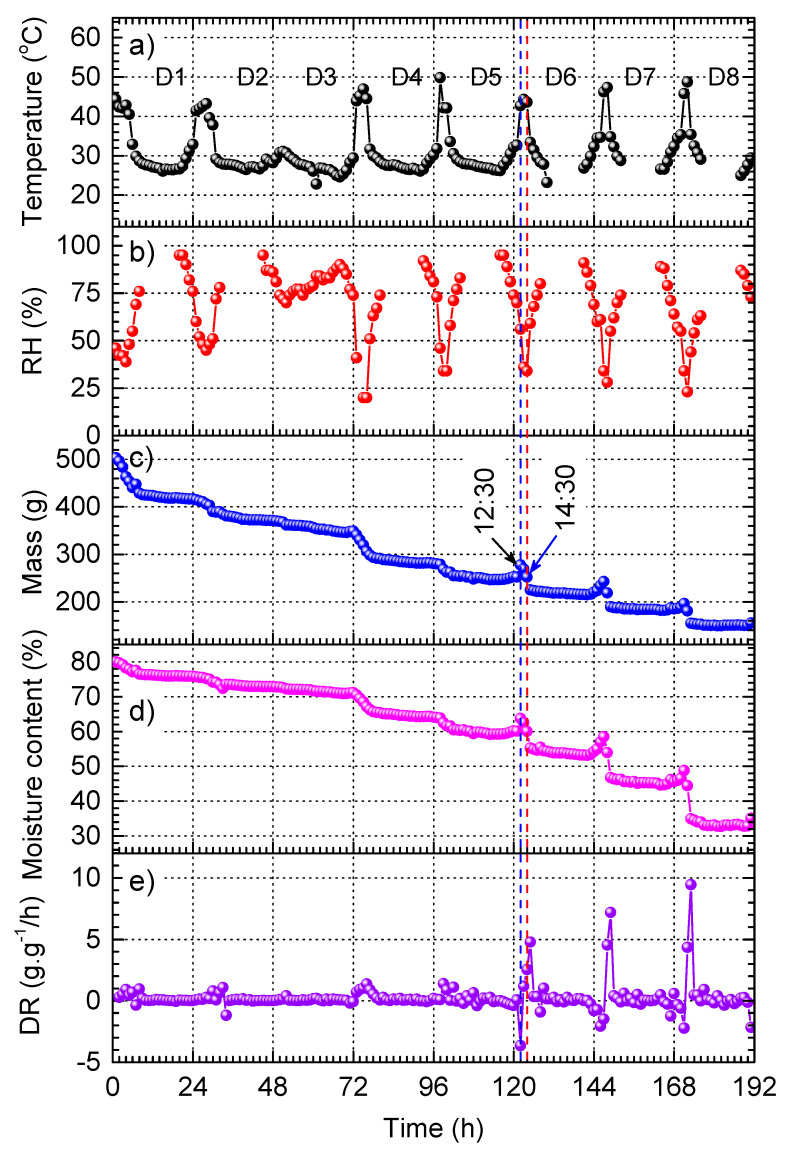
Open sun drying of red chilli: (**a**) ambient temperature, (**b**) ambient RH, (**c**) chilli mass, (**d**) moisture content, and (**e**) drying rate (DR) as functions of time acquired for 192 h.

Figure 4 shows the plots acquired for the open sun drying of red chilli, which was conducted for 8 consecutive days and nights (denoted as D1 to D8), starting from 10:30 AM of D1. The objective of this experiment is to investigate the influence of temperature and relative humidity (RH) on drying behavior. The temperature and RH as functions of time are plotted in Figure 4a and Figure 4b, respectively. The results demonstrate a daily periodic variation in both temperature and RH. The abnormal variation on D3 is due to the cloudy weather on that day. The results show a strong correlation between temperature and RH, in which temperature reaches its maximum and RH reaches its minimum between 11:00 AM and 16:00 PM.

The mass and the moisture content (wet basis) of red chilli as functions of time are shown in Figure 4c and Figure 4d, respectively. When the temperature increases to its peak (Figure 4a), the mass and moisture content are reduced with pronounced steps. Out of this period, both show a slight decrease, which is indicated by the insignificant drying rate shown in Figure 4e. The four plots in Figure 4b–e also reveal a very slow drying of red chilli in the whole D3, which is consistent with the low temperature and the high RH shown in Figure 4a and Figure 4b, respectively.

Interestingly, from D6 to D8, an anomalous effect repeatedly occurs during the peaking time of temperature. First, the mass rises rapidly, which is well synchronized with the increase in moisture content at the same time, resulting in a sudden drop in the drying rate (i.e., negative rate) shown in Figure 4e. It is important to note that when the mass and the moisture content reach their peaks (at 12:30), the temperature is still increasing. Thereafter, between 12:30 PM and 14:30 PM (visualized by the two dashed lines in Figure 4), the further increase in temperature results in a steep drop of the mass and the moisture content, causing a rapid increase in the drying rate (Figure 4e). The increases in mass and moisture content indicate that the re-absorption of moisture takes place even when the ambient temperature is still rising. Despite this phenomenon not being reported in literature, several studies suggested that red chilli are highly sensitive to moisture re-absorption [11,39,40].

After being dried to a certain level, the chilli surface may contain either a high density of pores or structural damages as reported in several studies [40,41,42,43]. The volume of pores can expand with increasing temperature, facilitating the absorption of moisture until an absorption–desorption equilibrium is reached. This can explain the increase in mass and moisture content. As the temperature continues to increase and RH continues to decrease, the desorption dominates, which consequently causes the reduction in mass and moisture content of red chilli. This is reflected by the rapid drop observed between 12:30 PM and 14:30 PM on D6–D8 shown in Figure 4c,d. The re-absorption prolongs the drying time and causes a negative impact on the quality of the dried products.

#### 3.3.2. Drying of Red Chilli in a Greenhouse (GH)

**Figure 5:**
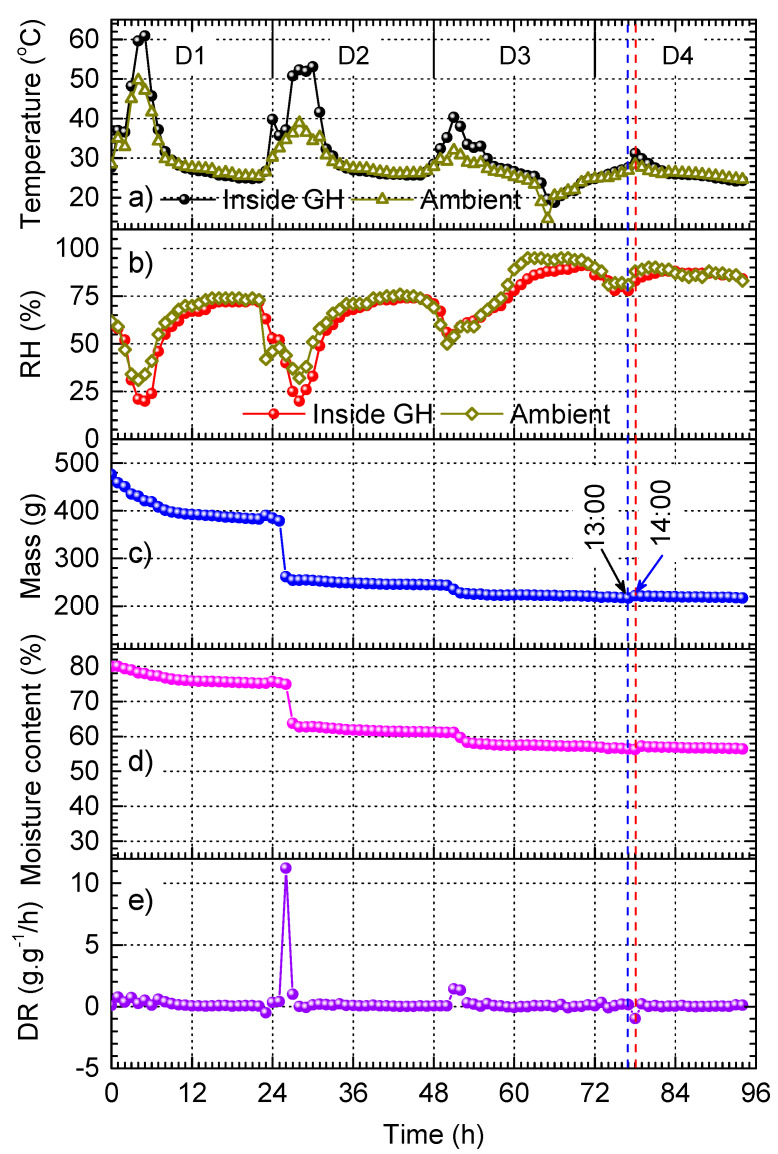
Drying of red chilli in GH: (**a**) temperature inside and outside of GH, (**b**) RH inside and outside of GH, (**c**) chilli mass, (**d**) moisture content, and (**e**) drying rate (DR) as functions of time acquired for 4 consecutive days.

Figure 5 shows the plots acquired for the drying of red chilli in a custom-built GH. The experiment was carried out in four consecutive days and nights (denoted as D1 to D4), starting from 8:00 AM of D1. We note that it was sunny on the first two days, and rainy and cloudy on the last two days. GH was installed in an open space so that sunlight was captured during the entire daytime. The temperature and RH inside GH and the ambience outside GH were monitored. The plots shown in Figure 5a,b reveal that the use of GH can reduce RH and increase the temperature inside it; both are beneficial for chilli drying.

The chilli mass and moisture content curves are shown in Figure 5c and Figure 5d, respectively. After the first day (D1), the chilli mass is reduced by 19.1%, whereas the moisture content is reduced by 4.3%. In comparison to the mass and moisture content decrease after one day of the experiment in open sun shown in Figure 4c,d, the decreases obtained for the GH experiment are slightly higher (17.1% for the mass and 4.1% for the moisture content decreases after D1 in Figure 4c,d). Remarkably, the drying efficiency on D2 is significantly more pronounced in GH. This is reflected by the emergence of the drying rate peak shown in Figure 5e, which reaches approximately 12 (g·g^−1^)·h^−1^. After D2, the chilli mass and the moisture content are reduced by 48.7% and 23.4%, respectively. These decreases are significantly higher than the corresponding numbers of 26.3% and 8.9% obtained for the open sun drying after the same drying time in Figure 4c,d. Taking into account the difference in temperature and RH, the results demonstrate that the use of GH can significantly enhance the drying efficiency of red chilli.

In addition, the nearly constant mass and moisture content observed for D3 and D4 in Figure 5 indicates that the drying does not take place due to the low temperature and high RH. Under the high RH condition, the re-adsorption may take place, which is indicated by a small increase in the chilli mass and the moisture content at the drying time of 77 h, which corresponds to the time at 13:00 PM on D4. Interestingly, this occurs when the temperature increases slightly. This small effect is consistent with the observation in Figure 4, which can support our hypothesis for the re-adsorption mechanism. Moreover, in both cases, we observe that the re-adsorption occurs when the moisture content is about 60%. Our observations provide hints of the origin of the re-adsorption, which could include three key parameters: temperature, ambient RH, and moisture content in the product. However, elaborate studies are needed to support these hypotheses.

#### 3.3.3. Drying of Red Chilli in GH with Black-Painted Plates

**Figure 6:**
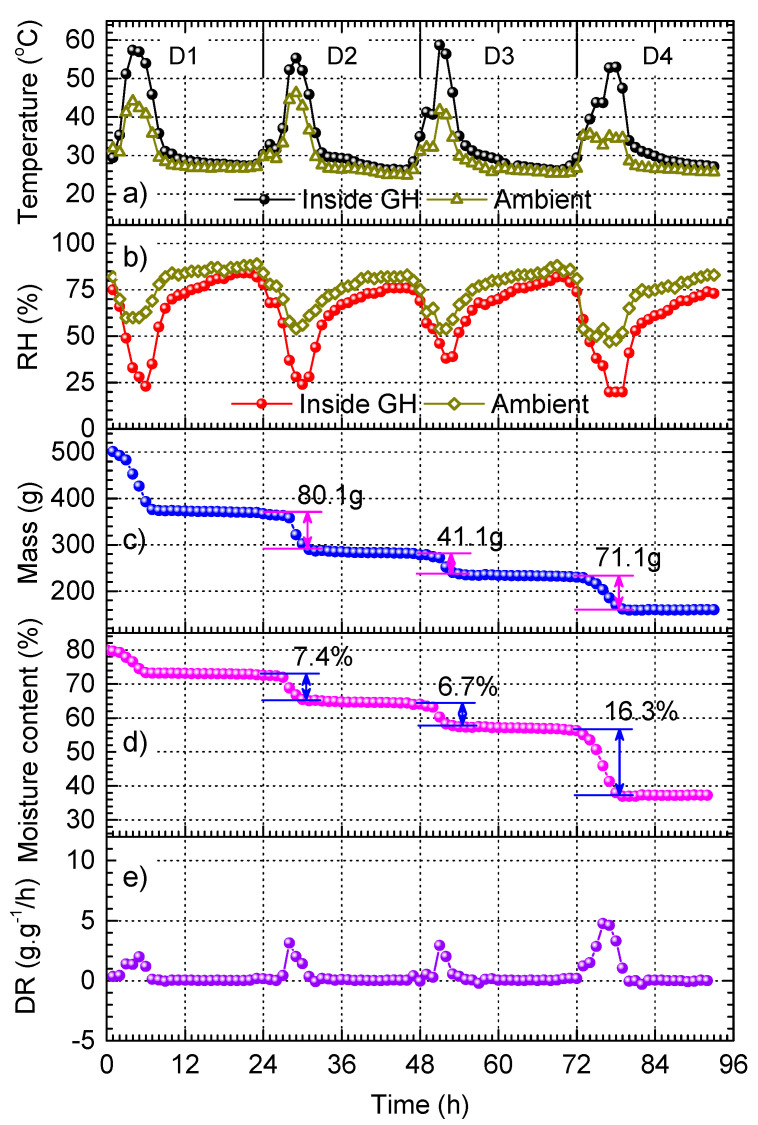
Drying of red chilli in p-GH: (**a**) Temperature inside and outside p-GH, (**b**) RH inside and outside GH, (**c**) chilli mass, (**d**) moisture content, and (**e**) drying rate (DR) as functions of time acquired for 92 h.

As we demonstrated in Section 3.2, black-painted metal plates are efficient substrates for sunlight harvesting and photothermal conversion. Therefore, we integrated these plates into GH (hereafter denoted as p-GH), which were attached to three walls of GH (see Figure 1 in the Materials and Methods section). Several previous studies demonstrated that the implementation of black-painted plates into the solar dryers increased remarkably the temperature inside it, which exhibited excellent performance in drying agricultural products, such as grapes [44] and strawberries [45]. Our experiment was carried out over approximately four consecutive days and nights (denoted as D1 to D4), starting from 8:30 AM of D1. The results are demonstrated in Figure 6. The temperature and RH graphs shown in Figure 6a,b indicate that the weather conditions were quite stable from D1 to D4, which provided ideal conditions for studying the drying behavior of red chilli.

The results demonstrate that p-GH offers great advantages for increasing the temperature and reducing RH. In particular, the highest ambient temperatures during the daytime on each day varies between 35 and 45 °C; however, inside p-GH, the temperature at the same time is always significantly higher, which can even reach 60 °C. Similarly, the lowest RH inside p-GH can reach 25%, whereas the lowest ambient RH is above 50%. According to the study of Getahun and Ebissa [18], the temperature and RH inside p-GH are in the optimum conditions for red chilli drying. Figure 6c,d shows that after approximately four days, the mass and the moisture content of red chilli are reduced by 68.1% and 53.4%, respectively. By comparing the mass loss in Figure 6c and the mass loss in Figure 4c (i.e., open solar drying) after the same drying time (i.e., 4 days), we can see that the drying time for the p-GH drying is approximately two times shorter. We note that in both cases, the ambient temperatures were in the same range. This indicates a great advantage of p-GH.

In addition, the analysis of decrease in mass and moisture content on each day (Figure 6c,d) reveals that the influence of RH on the drying of red chilli is more significant than temperature. For example, on D2 and D3, the temperature on D3 is slightly higher (Figure 6a); however, RH on D3 is higher (Figure 6b). The numbers presented in Figure 6c,d indicate that both the mass loss and moisture content drop are higher for D2. Similar results are obtained by comparing D3 and D4. This emphasizes the important role of RH, which contrasts to the common belief in the dominating role of temperature [4,21]. Furthermore, the re-adsorption effect is not observed for the entire drying process in p-GH. This could be considered as another advantage of using p-GH as a dryer for red chilli and other agricultural products.

#### 3.3.4. Drying of Red Chilli in GH with TiN-NPs-Embedded Black Paint

Despite the great advantage of saving energy by utilizing sunlight as the energy source, the use of GHs inherently possesses several major drawbacks, including a strong weather dependence and the discontinuous drying during the nighttime. These drawbacks are clearly demonstrated by the graphs shown in Figure 5 and Figure 6. We can see that, in all cases, the most effective drying time is between 10:00 AM and 16:00 PM. Out of this time range, the drying is insignificant. Hence, to improve the drying efficiency and shorten the drying time, an efficient continuous drying system is highly desirable.

To improve the nighttime drying, we implemented an additional heat source, which included a metal box made of Al-Zn alloy and a W-filament light bulb (50 W) installed inside the box. TiN NPs were mixed with black paint and coated onto the inner walls of the box. When the light bulb was switched on, the box was heated and became a heater. The role of TiN was to improve the photothermal conversion of the light generated by the bulb, as demonstrated in Figure 3b. We note that this heat source was only used for nighttime drying; hence, the light bulb was turned off during the daytime (from 8:00 AM to 17:00 PM). To demonstrate the efficiency of the design, the experiment was planned for sunny days based on the local weather forecast.

**Figure 7:**
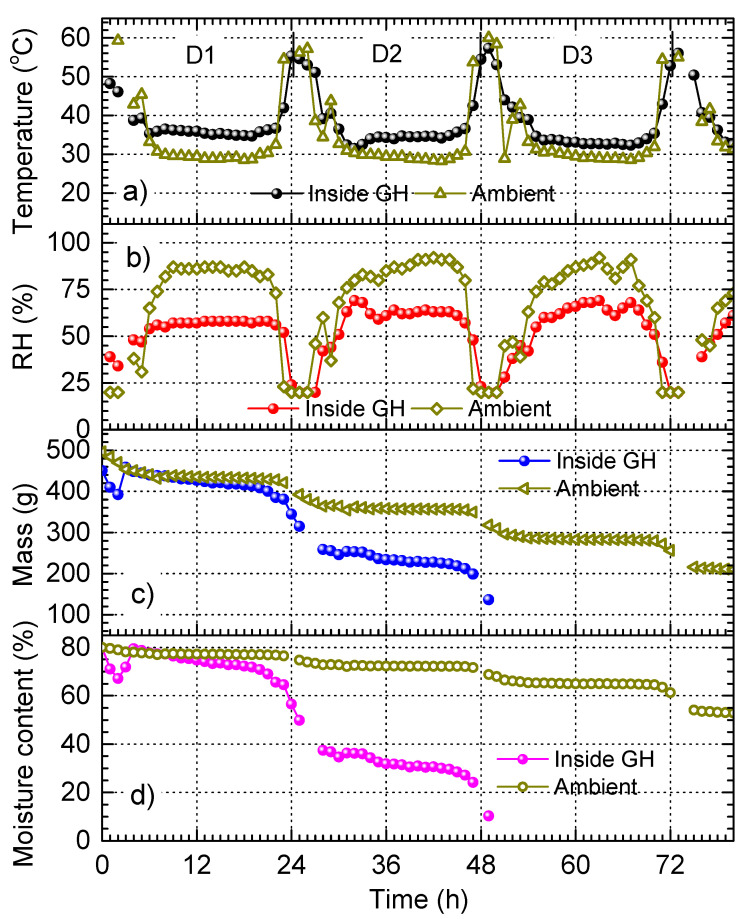
Drying of red chilli in GH with TiN NPs-embedded black painted box: (**a**) temperature inside and outside of GH, (**b**) RH inside and outside of GH, (**c**) chilli mass, and (**d**) moisture content as functions of time acquired for 80 h.

Figure 7 shows the graphs acquired for the drying of red chilli in GH with TiN NPs-embedded black painted box. The experiment was carried out for 80 consecutive hours, starting from 10:30 AM of D1. In this experiment, the drying of two baskets of chilli were monitored simultaneously; one basket was put next to the heat box, both of which were inside GH, the other one was put outside GH. The plots in Figure 7a indicate that when the lamp is switched off, no considerable difference in temperature is observed. This is in contrast with the temperature difference observed in Figure 5 and Figure 6 when comparing the temperature inside GH with the corresponding ambient temperature. This is because the ambient temperature in Figure 7a is the temperature inside the chilli layer measured by a thermocouple attached to the basket surface (i.e., not in the air as in other experiments). This temperature is significantly higher than the temperature in the air due to the heat trapping effect of the chilli layer. When the lamp is switched on, the temperature inside GH is clearly higher. In addition, the plots in Figure 7b show that RH inside GH is significantly lower. The results indicate that the use of the heat box improves both temperature and RH for drying red chilli.

The mass and the moisture content losses on D1 shown in Figure 7c,d are at the same level for the two chilli baskets. However, from D2, both mass and moisture content exhibit significant drops for the chilli inside GH. Within 2 days, the mass of the chilli in GH is reduced by 69.6%, resulting in the dried chilli with a moisture content of only 10.3%. For the open solar drying, in the same period of time, the mass is reduced by 35.9% and the moisture content remains at 68.8%. The results demonstrate that the drying efficiency can be remarkably enhanced by using the heat box. Particularly, GH enhances the mass reduction efficiency by a factor of 1.9, whereas an enhancing factor of 6.7 is achieved for the moisture removal efficiency. This is attributed to the significantly high photothermal conversion of the TiN NPs-embedded black paint as demonstrated in Figure 3b. Our results demonstrate another great potential application of TiN NPs.

#### 3.3.5. Quality of Dried Chilli by Different DRYING Methods

**Figure 8:**
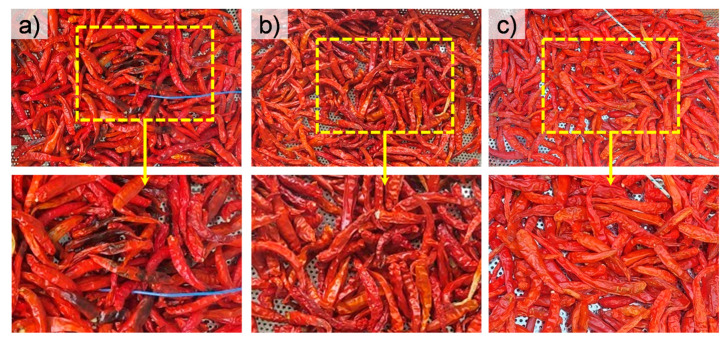
Photographs taken for the dried red chilli obtained by (**a**) open solar drying and (**b**) p-GH drying, and (**c**) GH drying with the heat box.

Figure 8 shows representative photographs of red chillies obtained by open solar drying (a), p-GH (b), and GH with TiN (c) as presented above. The bottom photographs show the areas marked by the dashed yellow rectangles in the top ones. It can be clearly seen that the open solar-dried products contain spoiled and fungal chilli. This could arise from the long drying time as well as the re-adsorption of moisture during the drying. Based on its appearance, we can see that the quality of the red chilli dried in GHs is significantly higher, especially for the one dried in GH with TiN NPs. Therefore, we can conclude that the use of GHs and the heat box boasts many advantages for practical applications in drying red chilli, which can be extended to the drying of other agricultural products.

## 4. Conclusions

In conclusion, we demonstrate that GHs using photothermal materials based on commercial black paint and TiN NPs can significantly improve the solar drying efficiency of red chilli, which increases the temperature, reduces the relative humidity, shortens the drying time, and results in a higher product quality. In particular, the use of TiN NPs can enhance the moisture removal efficiency by a factor of 6.7 and prevent the growth of fungi on the surface of dried chilli in comparison with the open sun drying process. The use of GHs also allows for investigating the influence of weather conditions on the drying mechanism. We found that the relative humidity can have a predominant role over the temperature in the solar drying of red chilli. In addition, the re-adsorption of moisture can take place during the drying process, which could be due to the thermal expansion of the pores on the chilli surface caused by the temperature increase. Our work demonstrates that the implementation of photothermal materials, such as TiN, brings many benefits that could lead to new developments in drying technology for agricultural products.

## Data Availability

The original contributions presented in the study are included in the article, further inquiries can be directed to the corresponding author.
